# Pneumonectomy in a child due to belated diagnosis of foreign body aspiration: a case report

**DOI:** 10.1186/s13256-021-03015-w

**Published:** 2021-10-20

**Authors:** Narindra N. M. Razafimanjato, Rindra A. Ralaivao, Tsiry D. N. Ravelomihary, Francis A. Hunald, Jean Louis H. Rakotovao

**Affiliations:** 1 Department of Surgery and Division of Thoracic Surgery, Faculty of Medecine, University Hospital of Joseph Ravoahangy Andrianavalona, University of Antananarivo, Antananarivo, Madagascar; 2 Department of Pathology, Faculty of Medecine, University Hospital of Joseph Ravoahangy Andrianavalona, University of Antananarivo, Antananarivo, Madagascar; 3 Department of Surgery and Division of Pediatrics Surgery, Faculty of Medecine, University Hospital of Joseph Ravoahangy Andrianavalona, University of Antananarivo, Antananarivo, Madagascar

**Keywords:** Foreign bodies, Pneumonia, Aspiration, Children, Morbidity, Mortality, Pneumonectomy

## Abstract

**Introduction:**

With early diagnosis, fiberoptic or rigid bronchoscopy methods are the gold standard in the management of tracheobronchial foreign body. Otherwise, nonrecognized bronchial foreign bodies cause irreversible damage to the airways and lungs. The deficiency of the health system noted in many developing countries such as Madagascar, combined with the fundamental problem relating to children’s conditions, which are determined by social and educational factors, makes it almost impossible to provide early and appropriate management of the penetration syndrome.

**Case presentation:**

An 11-year-old Malagasy female patient was referred to our hospital for an investigation of the etiology of the patient’s hemoptysis. The investigations revealed a localized bronchiectasis and atelectasis due to a foreign body obstructing the left main bronchus. Based on the hemoptysis and left lung almost destroyed by an occlusive lesion within, we decided to proceed with left pneumonectomy. A retrospective interrogation revealed a choking episode 4 years prior in elementary school after the child sucked on a pen cap and involuntarily aspirated it. Two years after the pneumonectomy, our patient was doing well and was asymptomatic.

**Conclusion:**

In this case report, we describe a rare case of a late presentation of foreign body aspiration that resulted in a left pneumonectomy in a child. Despite our favorable results, pneumonectomy must be the preferred last option. Preventive actions remain the optimal approach.

## Introduction

Intrabronchial foreign body aspiration (FBA) remains a serious accident that can be potentially fatal, mostly for children and the elderly [1]^2^. Interventional endoscopy techniques combined with advances in anesthesia have improved the conditions for its extraction. Although there is no standard guideline for managing FBA, bronchoscopic extraction is the first choice among all treatment modalities because of its low invasiveness in comparison with surgical treatments. In cases where the extraction would be unsuccessful by fiberoptic bronchoscopy, conventional surgery is needed to manage complications [3]. The deficiency of the health system noted in many developing countries such as Madagascar combined with the fundamental problem relating to children’s conditions, which are determined by social and educational factors, makes it almost impossible to provide early and appropriate management of the penetration syndrome. The delayed treatment of patients is sanctioned by an invasive and costly surgery leading to major parenchymal resection, which can be avoided in some occasions. In this case report, we describe a rare case of a late presentation of a foreign body aspiration that resulted in pneumonectomy in a child. With this case report, we would like to provide a contribution to the scientific literature by highlighting the serious lung problems caused by ABF in cases of delayed diagnosis.

## Case presentation

**Fig. 1:**
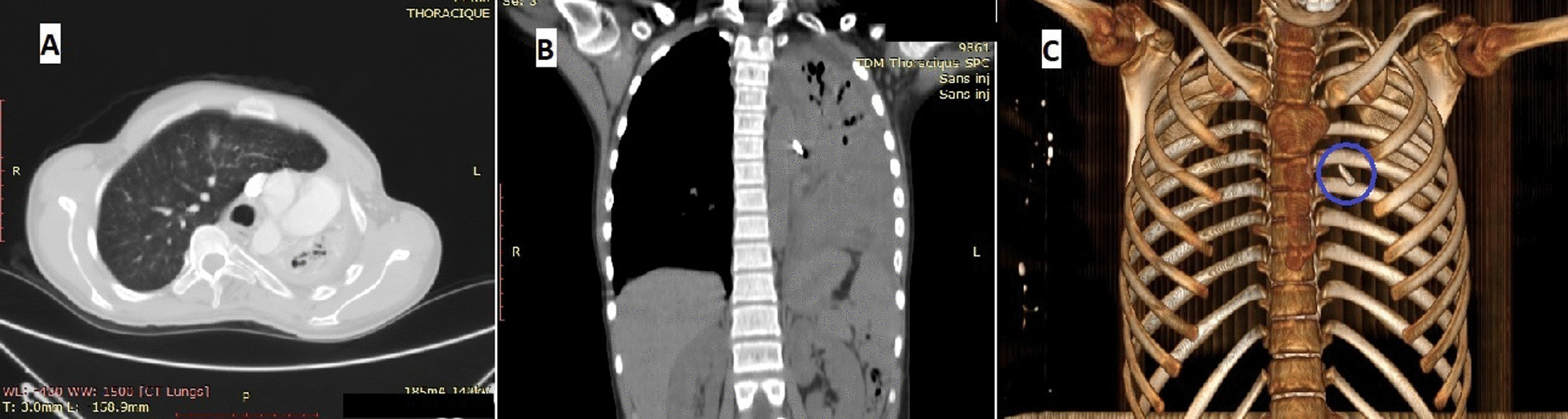
**A** Chest computed tomographic scan showing cavitary lesions in the left lung, as well as right lung herniates into the left chest. **B**, **C** Image of foreign body on thoracic computed tomography. The circle denotes a foreign body on thoracic computed tomography

**Fig. 2:**
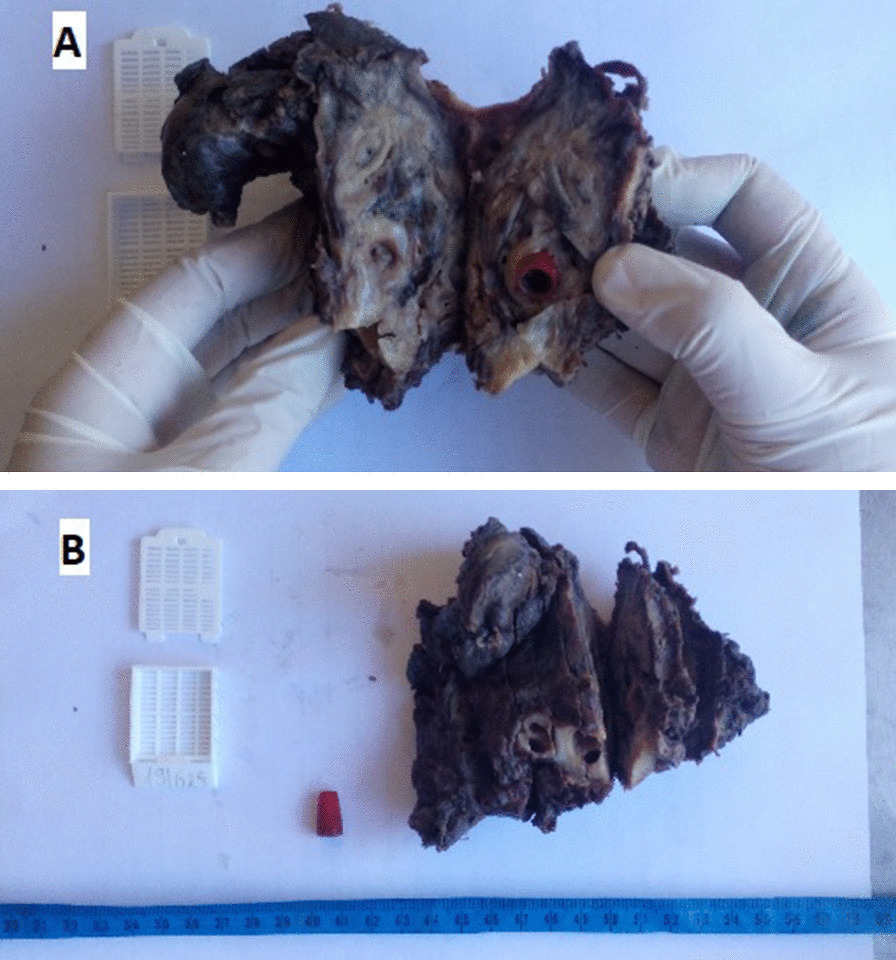
Definitive pathology result revealed, on sectioning the specimen. **A** Image of the left pneumonectomy material and **B** showing a red pen blind cap obstructing the left main bronchus, with distal bronchiectasis

An 11-year-old Malagasy female patient was referred to our hospital for investigation of productive cough with hemoptysis of bloody streaks. She had a 10-day history of fever and shortness of breath. In her detailed medical history, the patient was unaware of her accidental aspiration of the foreign body. Nevertheless, she had been diagnosed with prolonged and recurrent pneumonia symptom not responding to standard medical therapy. Clinically, the patient’s oxygen saturation was 96%, and she was spontaneously breathing (23 breaths/minute). Her heart rate was 90 beats/minute, with a stable hemodynamic status not presenting an altered level of consciousness (Glasgow score 15). Asymmetric chest wall movement was noted on physical examination. On auscultation, there were decreased breath sounds and signs of consolidation suggesting collapse of the pulmonary parenchyma of the left hemithorax. Chest X-ray showed changes consistent with bronchiectasis. Chest computed tomography showed localized bronchiectasis in the left upper lobe and atelectasis in the lower lobe, and revealed a foreign body obstructing the lumen of the left main bronchus with virtual bronchoscopy as well as a hernia of the right lung that invaded the left part, with a displacement of the mediastinal structures into the left hemithorax (Fig. 1). Based on hemoptysis and the left lung almost destroyed by the presence of an occlusive lesion within, a left pneumonectomy was decided, and she underwent a posterolateral thoracotomy. Intraoperatively, we could complete left pneumonectomy despite dense pleuropulmonary adherence. The patient was extubated safely after the surgery, and her oxygen saturation improved considerably. The postoperative period was uneventful with negative conversion of the clinicobiological inflammatory reaction. Our patient was discharged on the fifth postoperative day. The permanent pathologic result revealed, on sectioning the specimen, a red pen cap obstructing the left main bronchus, with distal bronchiectasis (Fig. 2). During a retrospective interrogation, she confessed about a choking episode in elementary school after she sucked the blind cap of her pen and involuntarily aspirated it 4 years ago. Two years after the pneumonectomy, our patient was doing well and was asymptomatic.

## Discussion

Destroyed lung is described as a nonfunctional lung and is most often caused by inflammatory diseases [4]. It is an unusual condition if it is due to foreign body aspiration. Foreign body aspiration occurs frequently in children because of their habit of placing objects in the mouth [5]. The consequences of their presence in the respiratory tract due to the delay in management are devastating, with very serious long-term respiratory disorders. According to the literature, an early extraction time limited to the first 72 hours after choking syndrome is an important condition for an uncomplicated outcome [3]. In our case, the foreign body was detected after 4 years. A systematic literature review reported that the duration of foreign body retention ranges from 18.4 to 25.8 months [6]. A long-standing foreign body in the bronchial tree results in irritation and inflammation and provokes the development of granulation tissue that causes obstruction. Any delayed obstruction of the bronchus and atelectasis can destroy the architecture of the lung and produce bronchiectasis and related complications such as hemoptysis, empyema, and recurrent pneumonia as presented in our observation [2] 5–8. If risk factors of unknown diagnosis such as alcohol use, drug abuse, senility, seizures, general anesthesia, trauma, mental retardation, and inhaled dentures are found in adults [5], we think that, in the Malagasy context with a strict formal education, we learned through observation that risk factors are due to the child’s inability to disclose a history of penetration syndrome because they fear their parents’ reactions. Early diagnosis of FBA is easy when the medical history of aspiration is evident or when the patients manifest definitive symptoms, such as choking [2]. As in our case, if the medical history is unclear and not conclusive, all attempts to confirm or to exclude the diagnosis must be made. On chest X-ray, not all intrabronchial foreign bodies are radiopaque [6]. The most common findings are radiological signs related to complications such as the destruction of the pulmonary parenchyma, atelectasis, hyperinflation, and bronchiectasis [2, 5, 6, 8–10]. Pulmonary destruction is often unilateral and localized and mainly affects the right lung owing to the anatomy of the right bronchus, which is approximately vertical [7]. In our case, the foreign body was localized in the left lung. In a study conducted by Nasim Tahir *et al*., the variability in the position of the carina with respect to the mid-trachea may explain why this right-sided preference is less pronounced in children compared with adults [11]. In delayed diagnosis, the bronchial wall in contact with the foreign body was hypertrophic with strong inflammation (granuloma formation, dense adhesion). In cases with delayed diagnosis (> 3 days), the incidence of granulomatous hyperplasia increases to 83.1% [12]. For these considerations, a standardized assessment of invasive bronchoscopy should be used to reduce unnecessary endoscopic procedures if extraction should be impractical. Some authors have reported on the success of belated removal of foreign body after the instillation of fibroblast inhibitory molecules [3]. The use of this technique may be justified if there are no arguments for bronchopulmonary complications, and especially if the delay in diagnosis is not significant. It was not the case in our observation because our center is neither equipped with pediatric fiberoptic bronchoscopy nor these molecules used, and our child presented with a destroyed left lung that was nonfunctional on CT scan. Surgical interventions are required in cases wherein bronchoscopic extraction is difficult, with the most frequent surgical strategy being a bronchotomy or a pulmonary resection [2]. As in literature, a surgical lung resection was required in our observation to address lung complications (the risk of contamination of the contralateral lung and to remove the shunt effects) to improve the patient’s quality of life [3–5]. However, children are at high risk of death, with possible major and minor morbidities following pneumonectomy, and we agree with the many authors in the literature that prudence is advised for this particular population of patients. In their follow-up period of evaluation of 18 cases of pneumonectomy, the authors reported no mortality and three postoperative minor complications. This study included various etiology of destroyed lung and concluded that the morbidity and mortality rates of pneumonectomy are acceptable for selected and well-prepared children [4]. In another evaluation, a study of pre- and postoperative characteristics of 20 children who underwent pneumonectomy for different etiologies showed that correct selection of indications, intensive preoperative rehabilitation with eradication of infection, careful execution of anesthetic and surgical procedures, early management of complications, and long-term support including pulmonary physiotherapy are essential conditions to decrease morbidity and mortality rates of pneumonectomy in child [9]. These risk factors for mortality in children following pneumonectomy were evaluated by Verónica Giubergia *et al.* in their study with 51 pneumonectomy in childhood, and the authors reported 4% mortality rate at 1 month. They concluded that age ≤ 3 years and the need for ventilation assistance ≥ 4 days were associated with increased risk of morbidity and mortality [13]. In the case of fatal aspiration of a foreign body, John Isherwood *et al.* reported two observations requiring extracorporeal membrane oxygenation (ECMO) support. This technique should be considered for patients with cardiorespiratory instability and who are then skeptical of bronchoscopy or early surgery [8].

## Conclusion

The inhalation of any foreign body can result in a serious and potentially fatal complication that warrants special attention from the parents, the educators, and the healthcare professionals. Though we notice favorable results in major resections for lungs destroyed by intrabronchial foreign bodies, pneumonectomy must be the preferred last option. Preventive actions remain the optimal approach. It is the most effective, cheapest, and least aggressive treatment and is therefore the best suited to our society for practical circumstances.

Take-home points

1. Children who suddenly start choking and coughing should draw their parents’ attention.
2. A complete medical history of the initial episode is very important in delayed FBA management.
3. In a country with TB endemicity such as Madagascar, radiological lung destruction should be part of the differential diagnosis of TB to avoid a delayed therapeutic decision.
4. Conventional surgery should be reserved for the case of unsuccessful extraction by bronchoscopy surgery as well as high risk of complications such as lung destruction.

## Data Availability

Not applicable.

## Abbreviations

FBA: Foreign body aspiration
ECMO: Extracorporeal membrane oxygenation
CT: Computed tomography
TB: Tuberculosis
